# The effectiveness of scenario-based virtual laboratory simulations to improve learning outcomes and scientific report writing skills

**DOI:** 10.1371/journal.pone.0277359

**Published:** 2022-11-11

**Authors:** Hakeemah Al-nakhle

**Affiliations:** Department of Medical Laboratories Technology, College of Applied Medical Sciences, Taibah University, Almadinah Almonawarah, Saudi Arabia; Central China Normal University, CHINA

## Abstract

The use of virtual laboratory simulations in various disciplines, which provide important educational benefits, has increased. Several studies show that laboratory activities, including scenario-based virtual laboratory simulation (SB-VLS), stimulate cognitive and non-cognitive skills. However, the effects of the SB-VLS when integrated into molecular biology courses, on the development of cognitive skills, such as scientific report writing skills, remain unexplored. A pre-post-test, randomized, quasi-experimental design was used. Thirty-five female students were randomly assigned to experimental or control groups. The control group (n = 17) attended a traditional lecture and video lab demonstration (VLD), while the experimental group (n = 18) participated in SB-VLS on molecular cloning. Findings revealed statistically significant differences, with large effects sizes in the SB-VLS group between pre- and post-test in intrinsic motivation (2.9 *vs* 3.86, *p = 0*.*042*, *Cohen’s d* = 4.17), self-efficacy (3.31 *vs* 3.85, *p = 0*.*002*, *Cohen’s d* = 1.071), and knowledge gain scales (50.93 *vs* 75.93, *p = 0*.*001*, *Cohen’s d* = 1.46). Moreover, between-group effect sizes of the experimental and control groups were also large for intrinsic motivation (*dppc2* = 1.441), self-efficacy (*dppc2* = 0.766), and knowledge (*dppc2* = 1.147), indicating that the effect of the SB-VLS was significant, which may be due to the activities and techniques used in SB-VLS to develop learning outcomes. Additionally, the SB-VLS group had statistically better lab report scores as compared to the control group (3.92 *vs*. 4.72, *p < 0*.*0001*). Collectively, our data show that SB-VLS is an innovative teaching strategy and an effective tool for developing non-cognitive and cognitive skills, especially scientific report writing skills.

## Introduction

Virtual lab simulations are being increasingly used to enhance the development of professional skills in various fields, such as healthcare [1] and education, including courses in chemistry [2, 3], biotechnology [4], and medical genetics [5]. Virtual learning simulations have also been applied to organs [6, 7] virtual dissections [8], and in human patient simulators [9]. Advantages of virtual laboratory simulations include cost-effectiveness, increased engagement of students with learning materials, and elimination of biosafety concerns [10]. They also allow students to observe the otherwise unobservable phenomena by reducing the time required to conduct experiments which require more time if conducted physically [10]. Learning through simulations provides students an opportunity to engage in inquiry-based learning that enables them to gain conceptual knowledge independently. Simulations also motivate and challenge students by providing continuous feedback in an environment that is tailored as per their individual interests and learning needs [11].

The molecular biology course in our medical laboratories technology program outlines the following five learning domains: knowledge, cognitive skills, interpersonal skills, responsibility, communication, and psychomotor skills. One of the course objectives is to develop cognitive skills, such as students’ abilities to interpret experimental outcomes and find justifications. I have observed a decline in students’ ability to justify their experimental findings through report writing.

In our molecular biology course, students’ laboratory reports do not indicate the depth of scientific writing skills, as they submit a report according to the laboratory manual without focusing on the techniques or employing analytical and communication skills. Students are not required to internalize content, justify findings, or master scientific practices in laboratory reports. This problem can be solved if laboratory exercises are integrated with interactive learning technologies containing problem-based scenarios, such as scenario-based virtual laboratory simulation (SB-VLS).

Improvement of scientific writing skills based on knowledge acquired from simulation can allow students to construct knowledge, relate it to prior information, and generate new findings, which can then be recorded in the scientific report. Indirectly, assessment using SB-VLS would benefit students in the final year as they would receive early research experience before their graduation project. Effective SB-VLSs direct students to questions, facilitate investigation of problems, and promote the process of inquiry. Furthermore, they allow students to apply creativity and critical thinking to find solutions to issues, including real-world problems.

An increasing body of evidence suggests that virtual laboratory simulations enhance learning outcomes by increasing students’ motivation and self-efficacy. Specifically, gamified laboratory simulations may facilitate better learning outcomes as compared to traditional teaching [4]. In preparing students for microbiology physical laboratory activities, virtual laboratory simulations are reportedly as efficient as face-to-face tutorials [12]. Furthermore, virtual laboratory simulations increase motivation and self-efficacy in students with low engagement [12]; integration of virtual laboratory simulations and physical laboratory activities is likely to improve students’ intrinsic motivation. Virtual learning simulations may provide positive learning experiences and hence are a valuable tool for developing cognitive and non-cognitive skills. This study examines motivation and self-efficacy as non-cognitive skills crucial in students’ academic achievement and improved writing abilities.

Students with higher self-efficacy show greater engagement and greater proficiency in completing writing tasks [13]. Additionally, they are more likely to participate actively and focus on the problem in case of a difficult learning task [13]. An increase in students’ self-efficacy and motivation improves writing abilities [14]. Consequently, one can posit that SB-VLS stimulates student’s self-efficacy and intrinsic motivation, thereby promoting knowledge acquisition and consequently, influencing their writing abilities when generating a scientific report.

It is critical for students who are studying molecular biology to be able to write and communicate scientific knowledge effectively to readers, and relate new information to previous knowledge fostering meaningful learning [15]. These skills are vital in order to present and justify scientific findings effectively. Such skills not only require searching for reliable information, but also justifying and discussing the results [16]. Writing not only facilitates deeper understanding of the subject but also aids in explicit learning [17].

Several studies have found that virtual lab simulations improve scientific writing skills, as evidenced in the lab reports [18–20]. Laboratory activities—particularly one employing inquiry-based laboratory learning strategy—can help students develop their scientific writing skills [21–24]. Indeed, students who use higher-order cognition in virtual laboratories exhibit better writing skills while preparing lab reports than students who use virtual laboratories for verification [22]. This improvement can be attributed to the active learning strategies associated with virtual laboratories that require higher-order cognition. In traditional laboratories, students report results on the basis of their observation without further analysis or without comparing their findings with the scientific literature.

In this study, cognitive skills were developed through SB-VLS, when students conducted a virtual experiment involving a problem, literature search, application of theories learned in lectures, and demonstration of critical thinking during problem solving. The non-cognitive skills focus on students’ motivation to improve their scientific writing and self-efficacy.

**Fig 1:**
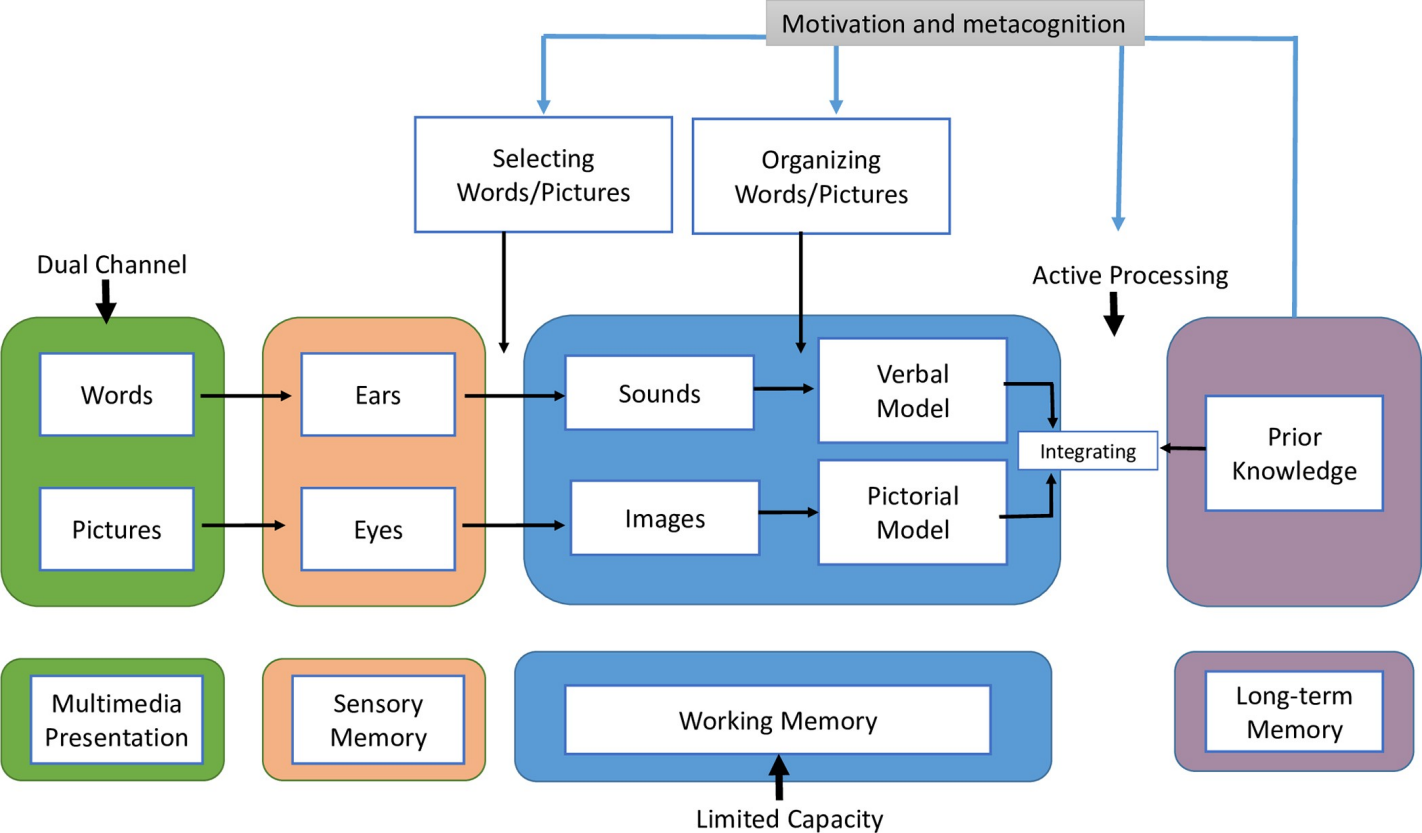
Visual representation of the components of the model in the cognitive theory of multimedia learning and their interactions with each other. Adapted from [25].

The cognitive theory of multimedia learning by Mayer [25] is used as a comprehensive theoretical framework to understand how virtual laboratory simulations may influence students’ learning outcomes, such as motivation and metacognition skills (Fig 1). The theory proposes a variety of constructs and mechanisms relevant to learning outcome development. This theory is based on three principles of information processing: first, dual processing: Learners process verbal and visual information differently. Second, limited capacity: Information can be stored only in a finite amount in the working memory. Finally, active processing: To acquire meaningful knowledge, learners must interact with information displayed in the simulation, organize it, use prior knowledge from the long-term memory and integrate it into their mental structures. As the learner actively processes information and strives to achieve the highest score on the quizzes embedded with the simulation, self-regulation skills, motivation, and self-efficacy are enhanced. Students gain metacognitive knowledge through learning about cognitive processes, i.e., learning how to control cognitive processes. Metacognition is the ability to reflect, conclude, and apply these conclusions in real-life situations [26]. Molecular biology experiment courses require metacognitive skills for solving problems related to experiments. In order to solve problems, students must be able to understand the problem, simulate models, perform experiments, and interpret and justify the solutions obtained in their lab reports [27].The specific objectives of this study were (i) to determine the effectiveness of using SB-VLS to enhance learning outcomes, including self-efficacy and intrinsic motivation, and (ii) to evaluate the impact of SB-VLS on improving knowledge and scientific report writing skills. In pursuance of the objectives, three research questions were formulated:

**Research question 1.** Does an SB-VLS lead to improvement intrinsic motivation and self-efficacy, related to the topic of molecular cloning?

**Research question 2.** Does an SB-VLS lead to improved student knowledge of the study topic, namely molecular cloning?

**Research question 3.** Does an SB-VLS lead to improved scientific report writing skills, as evidenced by improved lab report scores compared with a control group?

## Materials and methods

### Ethical approval

The Research Ethics Committee of the College of Applied Medical Sciences, Taibah University (2021/102/101/MLT), approved this study. Before the formal survey, all students were informed of the research objectives, and their consent was obtained. All participants’ data were kept confidential, and responses were kept anonymous.

### Participants, research design, sample size, and power analysis

Undergraduate medical laboratory technology students enrolled at the College of Applied Medical Sciences at Taibah University, Saudi Arabia, were recruited for this pilot study. The participants included 35 female students in the third year of their academic program in 2021. A pre-post-test, randomized, quasi-experimental design was used. The students who volunteered to participate were randomly assigned to experimental or control groups. An SB-VLS was used to develop cognitive skills (scientific writing skills and knowledge) and non-cognitive skills (self-efficacy and intrinsic motivation) of the experimental group (n = 18), while traditional instruction and video lab demonstration (VLD) were used in the control group (n = 17).

The magnitude of the SB-VLS effect was assessed based on the statistical significance and estimates of effect size. An effect size of 0.10–0.29 was considered small, 0.30–0.49 was considered moderate, and ≥ 0.50 was considered large, based on Cohen’s proposal for interpreting effect sizes [28, 29]. Additionally, post-hoc power analysis was performed using an online statistical program to determine whether the SB-VLS and VLD group sizes were sufficiently large to detect a statistical difference between the two dependent means, with an alpha level of 0.05 [28]. Moreover, the effect sizes (η^2^ and d for a non-parametric test) were calculated for the lab report score data (see S8 Table as reference for determination of the effect size η^2^ and d). The d_ppc2_ values were also calculated to estimate the effect size between groups. The d_ppc2_ values were interpreted according to the criteria suggested by Cohen (1988): a value from 0.2 to 0.5 indicated a small effect, 0.5 to 0.8 indicated a moderate effect, and greater than 0.8 indicated a large effect.

### Study instrument and measurement of the main outcomes

The questionnaire consisted of items that assessed the students’ knowledge (cognitive skills) and those that measured intrinsic motivation and self-efficacy (non-cognitive skills). Baseline knowledge of molecular cloning was assessed using six multiple-choice questions (**S1 Text**), and motivation was assessed using three questions adapted from the Interest/Enjoyment Scale of the Intrinsic Motivation Inventory [30] (**S1 Text**). Self-efficacy for learning molecular biology was assessed using eight questions adapted from the Motivated Strategies for Learning Questionnaire [31] (**S1 Text**). Students rated their responses to the motivation and self-efficacy items on a five-point Likert scale, ranging from 1 (Completely Disagree) to 5 (Completely Agree). An MCQ test comprising six items was administered to students in both groups to measure their knowledge (**S1 Text**).

The reliability and validity of the instrument that generated all study outcome variables for the pre- and post-test for both groups was estimated using Cronbach’s α coefficient, for which a value ≥ 0.70 represented good internal consistency of the items included in the questionnaire [32].

The effect of SB-VLS on the development of students’ scientific report writing skills was measured using lab report scores assigned to students in the VLD and SB-VLS groups.

### Procedure

Students were randomly divided into the control group (VLD) or experimental group (SB-VLS), and administered a pre-test to determine their baseline knowledge of molecular cloning, intrinsic motivation to study molecular biology, and self-efficacy; it also helped assess the equivalence of achievement between the two groups.

The VLD group received traditional learning for the practical portion (theoretical background and VLD). After completing traditional learning for 6 hrs on subjects relevant to molecular cloning, students were given a post-test to reassess their knowledge of molecular cloning, intrinsic motivation, and self-efficacy. Additionally, the students were asked to write a scientific lab report based on the lab manual, instructor’s instructions, VLD, and writing guidelines (**S2 Text**).

The SB-VLS session comprised 3 hrs of molecular cloning simulation followed by a 45 min post-test to estimate students’ cognitive and non-cognitive skills. The SB-VLS session takes less time than the VLD session because one of the advantages of the virtual labs is that they are able to provide more knowledge than traditional classes. This factor will not influence how we interpret the findings pertaining to group differences. Following the SB-VLS session, students were asked to write a scientific lab report to answer the research question, “Determine the function of radiation-sensitive 52 (*RAD52*) a DNA repair gene using molecular cloning techniques,” and to amalgamate their scientific findings from virtual experiments with their existing knowledge on relevant scientific literature. Students used the following learning tools to answer the research question: a laboratory manual provided by Labster, theoretical notes, and Labster video simulation related to molecular cloning. Students were allowed to repeat the simulations in their own time. They then collected and analyzed the data individually to explain and discuss their findings. This step investigated the effectiveness of the SB-VLS in improving scientific writing skills. The content of lab report for both groups included introduction, methods, results, discussion, and conclusions.

### Molecular cloning simulation scenario

Labster™ virtual simulation (molecular cloning case) was used in this study as an SB-VLS [33], as its simulation content aligns with the intended learning outcomes of molecular cloning topics, including DNA repair, mutation, and recombinant DNA technology. Students must choose individual protocols during the simulation, similar to real-life research. Furthermore, they can collect and interpret data before moving on to the next series of experiments. Students have access to experiments and molecular techniques which are unavailable in traditional undergraduate laboratories.

The simulation consisted of advanced laboratory equipment, including PCR and gel electrophoresis equipment, as well as 3D video animation; and learning tools, including theoretical and background information. For instance, students can visualize DNA duplication during the PCR process (which cannot be visualized in traditional laboratories) and how DNA samples run in gel electrophoresis and turn into DNA profiles. Additionally, students can work with a realistic case containing a central problem. They can utilize the equipment available in the virtual laboratory to solve a problem or answer research questions. While progressing through the simulation, students can learn concepts and techniques relevant to the case problem. They respond to MCQs throughout the simulation to assess whether they learned the concept; they cannot move to the next step until they answer all questions correctly [11].

Molecular cloning simulation introduces students to a scenario in which a researcher examines the function of RAD52 protein and its role in DNA repair. During virtual experiments, students can examine the function of RAD52, a DNA repair gene, and recognize several molecular cloning techniques, including DNA extraction and preparation, ligation, transformation, plate streaking, and antibiotic selection. The scenarios enable students to learn how to assemble an expression vector containing a specific regulator (TetOff) of the *RAD52*, and *GFP* genes and control the expression level of *RAD52* using doxycycline.

### Rubrics for assessing scientific report writing skills

The data score for scientific report writing skills was obtained by assessing scientific lab reports from both groups (VLD and SB-VLS). All reports were graded based on the same criteria, and each section (introduction, methods, results, discussion, and conclusion) was evaluated independently based on a scoring rubric (S7 Table) adapted from [34]. The total score out of 50 was divided by 10 to give a final score out of 5. All lab reports were blind marked by one tutor.

### Data analysis

The data collected during the research were analyzed using Graph Pad Prism 5.0 (Graph Pad Software, Inc., San Diego, CA). The construct reliability was determined using Cronbach’s alpha to estimate whether the instrument consisting of a multiple Likert-scale items was reliable. A Cronbach’s alpha value ≥ 0.70 is generally accepted as indicating good internal consistency in most social science research studies [32]. The paired t-test was used to determine if any differences existed within groups (between pre-test and post-test). The unpaired t-test was used to determine if any differences existed between the groups. The Kolmogorov–Smirnov test was used to determine the normality. The Mann–Whitney *U* test for unpaired samples was used to compare the lab report scores. Cohen’s d for pre-versus post-treatment effect sizes within groups was calculated. To adjust the baseline measurements, the effect size dppc2 was computed for the pre-post comparison of the experimental and control groups [35].

## Results

Means and standard deviations (SDs) of pre- and post-test scores for the three outcome variables (knowledge of molecular cloning, intrinsic motivation, and self-efficacy) are illustrated in Table 1 for both groups.

**Table 1:** Comparisons of intrinsic motivation, self-efficacy, and knowledge between the SB-VLS and control groups (VLD).

Scale/group	n	Pre-test M (SD)	Post-test M (SD)	*p-value*	Estimate (95% CI)	t-test t, df	Power	Pre-Post within group effect sizes (Cohen’s *d*)	Between-group effect sizes *d*_*ppc2*_	Cronbach’s alpha	
										Pre-test	Post-test
**Intrinsic motivation**											
**Experimental (SB-VLS)**	18	2.902 (0.3240)	3.863 (0.03396)	**0.0425**	-0.9608 (-1.842 to -0.07991)	t = 4.693 df = 2	1	4.171774 large	**1.441** large	0.821846	0.730803
**Control (VLD)**	17	2.804 (0.4173)	3.216 (0.5652)	0.5189	-0.4118 (-2.695 to 1.871)	t = 0.776 df = 2	**0.473688**	**0.829333** large		0.809748	0.8125
**Self-efficacy**											
**Experimental (SB-VLS)**	18	3.331 (0.561)	3.846 (0.385)	**0.0021**	-0.5147 (-0.7706 to -0.2588)	t = 4.757 df = 7	**0.702196**	**1.070946** large	**0.766** large	0.782259	0.709031
**Control (VLD)**	17	3.162 (0.5462)	3.243 (0.4200)	0.2518	-0.08088 (-0.2340 to 0.07228)	t = 1.249 df = 7	0.091179	0.163533 small		0.717999	0.721471
**Knowledge-based questions**											
**Experimental (SB-VLS)**	18	50.93 (18.40)	75.93 (15.58)	**0.0011**	-25 (-34.58 to -15.42)	*t = 6*.*708* *df = 5*	**0.908780**	**1.466** large	**1.147** large	–	–
**Control (VLD)**	17	49.02 (15.64)	53.92 (14.61)	0.1412	-4.902 (-12.12 to 2.316)	t = 1.746 df = 5	0.151967	0.324 small		–	–

*Note*. mean; SD, standard deviation; df, degrees of freedom; CI, confidence interval; SB-VLS, scenario-based virtual laboratory simulation; VLD, video lab demonstration; d_ppc2_, pre-test-post-test control group design

As shown in Table 1, paired *t*-tests were used for each group to estimate if a significant increase in knowledge pertaining to molecular cloning, intrinsic motivation, and self-efficacy occurred from the pre-to post-test. For the SB-VLS group, a significant increase in knowledge was detected from a mean of 50.93% correct responses on the pre-test to 75.93% correct responses on the post-test, (t (18) = 6.708, p = 0.001, df = 5) (S5 Table). Post-hoc power analysis for the SB-VLS group for the knowledge gain scale revealed that a sample of 18 students provided a power of 91% for detecting statistical differences for the effect size given by the mean scores. A significant increase in intrinsic motivation was found, from a mean of 2.902 on a scale of 1 to 5 in the pre-test to 3.863 in the post-test (t (18) = 4.693, p = 0.0425, df = 2) (S4B and S4D Table). Moreover, post-hoc power analysis of the intrinsic motivation scale revealed that a sample of 18 students provided a power of 100% for detecting statistical differences for the effect size given by the mean scores. Finally, self-efficacy increased significantly in this group, from a mean of 3.331 in the pre-test to 3.846 in the post-test (t (18) = 4.757, p = 0.0021, df = 7) (S3B and S3D Table). Post-hoc power analysis revealed the sample size of this group to be inadequate, with a statistical power of over 70% (according to S9 Table, the study should have at least 80% power to detect the intervention effect).

For the VLD group, paired sample t-tests showed no significant differences in knowledge gain scale, intrinsic motivation, or self-efficacy.

Table 1 also reveals a large effect size within the SB-VLS group for intrinsic motivation (Cohen’s *d* = 4.17), self-efficacy (Cohen’s *d* = 1.071) and knowledge (Cohen’s *d* = 1.46). Between-group effect sizes of the experimental and control groups were also large for intrinsic motivation (*d*_*ppc2*_ = 1.441), self-efficacy (*d*_*ppc2*_ = 0.766) and knowledge (*d*_*ppc2*_ = 1.147), indicating that the effect of the SB-VLS was significant, which may be due to the activities and techniques used in the SB-VLS to develop learning outcomes.

Additionally, the degree of internal reliability of the two outcome measures (intrinsic motivation and self-efficacy scales) in the pre- and post-test for both groups were evaluated by Cronbach’s alpha. As shown in Table 1, for the SB-VLS group, the Cronbach’s alpha reliability coefficient of the intrinsic motivation scale was 0.82 for the pre-test and 0.73 for the post-test, while for the self-efficacy scale, it was 0.78 and 0.71, respectively (S3A and S3C, S4A and S4C Tables). For the VLD group, the Cronbach’s alpha reliability coefficient of the intrinsic motivation scale was 0.809 for the pre-test and 0.8125 for the post-test, while for the self-efficacy scale, it was 0. 717 and 0.721, respectively (S1A and S1C, S2A and S2C Tables).

### Controlling possible confounding variables

To ensure that the participants were equivalent in their scores in the possible confounding variables, a pre-test was administered to both the groups. Pre-tests can assess variables that might influence the outcomes of the study and avoid any possible external interference. An independent sample t-test was used to compare means between experimental and control groups for all study variables. Table 2 shows the mean and the SD of each group in the pre-test. The analysis indicated that no statistically significant differences were present between the experimental and control groups. Furthermore, the means and SDs of both groups on the post-test were calculated using independent sample t-tests to assess the significance of any differences. Table 3 shows that statistically significant differences were present between the experimental and control groups for self-efficacy and knowledge at post-test, whereas no significant differences were observed between the experimental and control groups in intrinsic motivation at post-test.

**Table 2:** Independent sample t-test results between the experimental and control groups for the pre-cognitive and non-cognitive skills test.

Skills	Group	N	Mean	SD	t-test t, df	*p-value*
**Intrinsic motivation**	Experimental	18	2.902	0.3240	t = 0.3214 df = 4	0.7640
	Control	17	2.804	0.4173		
**Self-efficacy**	Experimental	18	3.331	0.5462	t = 0.6111 df = 13	0.5509
	Control	17	3.162	0.5606		
**Knowledge-based questions**	Experimental	18	50.93	18.40	t = 0.1934 df = 10	0.8505
	Control	17	49.02	15.64		

**Table 3:** Independent sample t-test results between the experimental and control groups for the post cognitive and non-cognitive skills test.

Skills	Group	N	Mean	SD	t-test t, df	*p-value*
**Intrinsic motivation**	Experimental	18	3.863	0.03396	t = 1.979 df = 4	0.1189
	Control	17	3.216	0.5652		
**Self-efficacy**	Experimental	18	3.846	0.3850	t = 2.993 df = 14	0.0097
	Control	17	3.243	0.4200		
**Knowledge-based questions**	Experimental	18	75.93	15.58	t = 1.746 df = 5	0.0302
	Control	17	53.92	14.61		

### Assessment of scientific report writing skills

Descriptive statistics for the grades assigned to lab reports for the control and experimental groups are shown in Table 4. The mean scores for the control and experimental groups were 3.918 (SD = 0.5753) and 4.72 (SD = 0.34), respectively (S6 Table). A significant difference was observed in the mean scores between the groups. The results showed that students in the SB-VLS group obtained higher average scores on the scientific lab reports as compared with the VLD group.

**Table 4:** Non-parametric comparison of student laboratory report scores between the VLD and SB-VLS groups.

Scale	VLD N = 17 group	SB-VLS N = 18 group	Comparison (Mann–Whitney U)	Eta squared (*η*^*2*^)	Effect size (Cohen’s *d*)
	Mean (SD)	Mean (SD)			
Lab report score	3.918 (0.5753)	4.72 (0.3418)	*U* = 34.5, *p* < 0.0001	0.437 large	1.762 Large

Table 4 shows that the effect size of the SB-VLS was large, indicating that the effect of the SB-VLS was pronounced, which may be due to the activities and techniques used in the SB-VLS to develop students’ scientific report writing skills.

## Discussion

This study found that SB-VLS could be a good formative assessment tool for evaluating learning outcomes, particularly cognitive and non-cognitive skills, among medical laboratory technology students. Our findings revealed that the knowledge level, scientific report writing skills, self-efficacy, and intrinsic motivation increased significantly in the experimental group as compared to the control group with respect to the topic “Molecular Cloning”, suggesting that engaging in simulation-based learning activities can lead to positive cognitive and non-cognitive outcomes.

The development of cognitive skills, such as knowledge and scientific report writing skills, is a crucial learning outcome and the main objective of an educational activity. However, growing evidence suggests that non-cognitive skills are also crucial in academic success [36]. Increased self-efficacy is associated with greater educational and life outcomes [13]. Furthermore, intrinsic motivation is essential as a mediator of short- and long-term educational outcomes [37].

The first research question asked if SB-VLS would influence non-cognitive skills. Our findings revealed a significant increase in self-efficacy and intrinsic motivation compared with traditional learning. These results are consistent with the previous findings [4, 12]. Therefore, it is evident that the SB-VLS approach is more effective than traditional learning in developing students’ self-efficacy and intrinsic motivation toward molecular biology. Indeed, improving non-cognitive skills significantly influences educational outcomes and academic success. Furthermore, students with high self-efficacy achieve complex tasks, enjoy challenges, work harder, and have a greater commitment to goals [5].

The second research question explored whether the SB-VLS affected students’ cognitive skills. Knowledge gain was compared through pre- and post-tests; results indicated a significant increase in knowledge when the students were exposed to the SB-VLS compared with the VLD, suggesting that the SB-VLS promotes knowledge gain. These results align with other studies’ findings that virtual laboratory simulation increases knowledge and the level of understanding [4].

The third research question examined integrating the SB-VLS into molecular biology courses to improve students’ scientific report writing skills. The students’ achievements in scientific report writing were evaluated based on their ability to write a lab report, and find information, comprehend it, and build critical thinking in the process of justifying their experimental findings. The results showed that students who used the SB-VLS approach had better average scores than the control group in the lab reports, suggesting that the former had a positive effect on scientific report writing skills.

Scientific report writing skills can be developed when students engage in a virtual experiment that answers research questions, allowing them to apply the knowledge taught through lectures to real-life situations to solve problems. The SB-VLS engages students with real-life tasks. Consequently, as students conduct authentic investigations, they become more involved in the writing process—particularly persuasive writing—when they contribute to finding solutions to real problems. This teaching strategy enables students to communicate scientifically while thoroughly understanding the problem and cause-effect relationships [22]. We, therefore, speculate that students who rely on the VLD approach are unable to improve their scientific report writing skills. Moreover, the effect size within the SB-VLS group was large, the effect of the SB-VLS was significant as the activities and techniques of the strategy contributed to the development of scientific report writing skills. These activities included an interactive research scenario that brought science to life, embedded quizzes, and video animations. It is encouraging to compare our findings with those of Sapriadil et al. [22], who reported that the scientific writing skills of students using higher-order thinking virtual laboratories were higher than those of students in a lab implementation class. Thus, our collective findings highlight the success of SB-VLS as an approach for improving students’ scientific report writing skills and learning outcomes. Moreover, this approach serves to enhance students’ critical thinking, creativity, conceptual understanding skills, motivation, and subject interest.

## Conclusion and future recommendations

The SB-VLS is an innovative teaching strategy that provides educational benefits and improves cognitive and non-cognitive skills. Our findings confirmed that the SB-VLS approach can be a valuable tool for improving students’ scientific report writing skills by utilizing the unique features of virtual lab simulations. This improvement was associated with strengthening students’ self-efficacy, intrinsic motivation, and knowledge.

SB-VLS may enhance several learning skills, including problem-solving skills, critical thinking, creativity, conceptual understanding, science process skills, motivation, interest, and help attain better learning outcomes, leading to improved scientific report writing skills. Studying SB-VLS’s impact on scientific report writing skills in the future research can be accomplished by measuring these learning skills. As part of students training programs in medical laboratories’ technology programs, they could be expected to be able to apply SB-VLS customized to their curriculum to develop their scientific report writing skills.

### Study limitations

The primary limitation of the current study was the small number of participants. Therefore, it is necessary to perform additional studies with a larger population to facilitate the generalization of these findings and for verifying the current results. Furthermore, only female students were included due to time limitations; there was insufficient time available to include male students, who study on a separate campus. Thus, future studies must incorporate participants from other genders.

## Supporting information

S1 Text The questionnaire used in the study for intrinsic motivation, self-efficacy, and knowledge. (DOCX)

S2 Text Guidance for writing lab reports. (DOCX)

S1 Table A-D. Percentage of student responses and Cronbach’s alpha calculation of student responses on the questionnaire of the self-efficacy (pre and post-test /control group).
(DOCX)

S2 Table A-D. Percentage of student responses and Cronbach’s alpha calculation of student responses on the questionnaire of the academic intrinsic motivation (pre and post-test /control group).
(DOCX)

S3 Table A-D. Percentage of student responses and Cronbach’s alpha calculation of student responses on the questionnaire of the self-efficacy (pre and post-test / experimental group).
(DOCX)

S4 Table A-D. Percentage of student responses and Cronbach’s alpha calculation of student responses on the questionnaire of the academic intrinsic motivation (pre and post-test / experimental group).
(DOCX)

S5 Table Comparisons in average % correct responses for knowledge scale (pre-posttest) for the SB-VLS and the VLD groups. (DOCX)

S6 Table Lab report scores results. (DOCX)

S7 Table Lab report grading rubric. (DOCX)

S8 Table Reference to determine the Level of size Effect (^2^η) and (d). (DOCX)

S9 Table Power by effect size. (DOCX)

## References

[1] Issenberg SB, McGaghie WC, Hart IR, Mayer JW, Felner JN, Petrusa ER, et al. Simulation technology for health care professional skills training and assessment. JAMA. 1999;282: 861–866. doi: 10.1001/jama.282.9.861 10478693

[2] Plass JL, Milne C, Homer BD, Schwartz RN, Hayward EO, Jordan T, et al. Investigating the effectiveness of computer simulations for chemistry learning. J Res Sci Teach. 2012;49: 394–419. doi: 10.1002/tea.21008

[3] Yaron D, Karabinos M, Lange D, Greeno JG, Leinhardt G. SPORE series winner. The ChemCollective—virtual labs for introductory chemistry courses. Science. 2010;328: 584–585. doi: 10.1126/science.1182435 20431007

[4] Bonde MT, Makransky G, Wandall J, Larsen MV, Morsing M, Jarmer H, et al. Improving biotech education through gamified laboratory simulations. Nat Biotechnol. 2014;32: 694–697. doi: 10.1038/nbt.2955 25004234

[5] Makransky G, Bonde MT, Wulff JS, Wandall J, Hood M, Creed PA, et al. Simulation based virtual learning environment in medical genetics counseling: an example of bridging the gap between theory and practice in medical education. BMC Med Educ. 2016;16: 98. doi: 10.1186/s12909-016-0620-6 27012245 PMC4807545

[6] Av-Ron E, Byrne JH, Baxter DA. Teaching basic principles of neuroscience with computer simulations. J Undergrad Neurosci Educ. 2006;4: A40–52. 23493644 PMC3592631

[7] Davis MJ, Gore RW. Determinants of cardiac function: simulation of a dynamic cardiac pump for physiology instruction. Adv Physiol Educ. 2001;25: 13–35. doi: 10.1152/advances.2001.25.1.13 11824189

[8] Predavec M. Evaluation of E-Rat, computer-based rat dissection, in terms of student learning outcomes. J Biol Educ. 2001;35: 75–80. doi: 10.1080/00219266.2000.9655746

[9] Cesari WA, Caruso DM, Zyka EL, Schroff ST, Evans CH Jr, Hyatt JP. Study of physiological responses to acute carbon monoxide exposure with a human patient simulator. Adv Physiol Educ. 2006;30: 242–247. doi: 10.1152/advan.00063.2006 17108253

[10] Ton de J, C. LM, C. ZZ. Physical and virtual laboratories in science and engineering education. Science. 2013; 340:305–308. doi: 10.1126/science.1230579 23599479

[11] Thisgaard M, Makransky G. Virtual learning simulations in high school: effects on cognitive and non-cognitive outcomes and implications on the development of STEM academic and career choice. Front Psychol. 2017; 8: 805. doi: 10.3389/fpsyg.2017.00805 28611701 PMC5447738

[12] Makransky G, Thisgaard MW, Gadegaard H. Virtual simulations as preparation for lab exercises: assessing learning of key laboratory skills in microbiology and improvement of essential non-cognitive skills. PLoS One. 2016;11: e0155895. doi: 10.1371/journal.pone.0155895 27253395 PMC4890735

[13] Zimmerman. Self-efficacy: An essential motive to learn. Contemp Educ Psychol. 2000;25 1:82–91. doi: 10.1006/ceps.1999.1016 10620383

[14] Yulianawati I. The role of self-efficacy in students’ writing ability (A case study at second grade students of a senior high school in Indramayu). Vis J Lang Foreign Lang Learn. 2019;8:79. doi: 10.21580/vjv8i13470

[15] Prain V. Learning from Writing in Secondary Science: Some theoretical and practical implications. Int J Sci Educ. 2006;28:179–201. doi: 10.1080/09500690500336643

[16] Weaver CL, Duran EC, Nikles JA. An Integrated Approach for Development of Scientific Writing Skills in Undergraduate Organic Lab. In: Addressing the Millennial Student in Undergraduate Chemistry. Vol 1180. ACS Symposium Series. American Chemical Society; 2014:105–123 SE—8. doi: 10.1021/bk-2014-1180.ch008

[17] Watts FM, Spencer JL, Shultz G V. Writing assignments to support the learning goals of a CURE. J Chem Educ. 2021;98:510.

[18] Finkelstein ND, Adams WK, Keller CJ, et al. When learning about the real world is better done virtually: A study of substituting computer simulations for laboratory equipment. Phys Rev ST Phys Educ Res. 2005;1:10103. doi: 10.1103/PhysRevSTPER.1.010103

[19] Jillian L. Comparative study of hands-on and remote physics labs for first year university level physics students. Transform Dialogues Teach Learn Journal. 2012;6:1–25.

[20] Brinson JR. Learning outcome achievement in non-traditional (virtual and remote) versus traditional (hands-on) laboratories: A review of the empirical research. Comput Educ. 2015;87:218–237. doi: 10.1016/j.compedu.2015.07.003

[21] Malik A, Setiawan A, Suhandi A, Permanasari A. Enhancing pre-service physics teachers’ creative thinking skills through HOT lab design. AIP Conf Proc. 2017;1868:70001. doi: 10.1063/1.4995177

[22] Sapriadil A, Setiawan A, Suhandi A, Malik D, Safitri SASL, NH. Optimizing students’ scientific communication skills through higher order thinking virtual laboratory (HOTVL). J Phys Conf Ser. 2018; 1013(3 012050).

[23] Alpusari M, Mulyani EA, Putra ZH, Widyanthi A, Hermita N. Identifying students’ scientific communication skills on vertebrata organs. J Phys Conf Ser. 2019;1351:12070. doi: 10.1088/1742-6596/1351/1/012070

[24] Aydın G. Impacts of inquiry-based laboratory experiments on prospective teachers’ communication skills. Int Online J Educ Sci. 2016;8. doi: 10.15345/iojes.2016.02.005

[25] Mayer RE. In: Mayer RE, editor. The Cambridge Handbook of Multimedia Learning. 2nd ed. Cambridge: Cambridge University Press; 2014. p. iii-iii. (Cambridge Handbooks in Psychology). doi: 10.1017/CBO9781139547369

[26] Amin I, Mariani S. PME Learning Model: The Conceptual Theoretical Study Of Metacognition Learning In Mathematics Problem Solving Based On Constructivism. INT ELECT J MATH ED. 2017;12:333–52. doi: 10.29333/iejme/616

[27] Ongowo R, Indoshi F. Science Process Skills in the Kenya Certificate of Secondary Education Biology Practical Examinations. Creative Education. 2013; 4: 713–717. doi: 10.4236/ce.2013.411101

[28] Soper DS. Post-hoc Statistical Power Calculator for a Student t-Test [Software]. https://www.danielsoper.com/statcalc. Published 2022.

[29] Cohen J. Statistical Power Analysis for the Behavioral Sciences (2nd Edition). Hillsdale, NJ: Lawrence Earlbaum Associates.; 1988.

[30] Deci EL, Eghrari H, Patrick BC, Leone DR. Facilitating internalization: the self-determination theory perspective. J Pers. 1994; 62:119–142. doi: 10.1111/j.1467-6494.1994.tb00797.x 8169757

[31] Pintrich P., Smith D., García T., & McKeachie W. A Manual for the Use of the Motivated Strategies for Learning Questionnaire (MSLQ). 1991.

[32] Quansah F. The use of Cronbach alpha reliability estimate in research among students in public universities in Ghana. African J Teach Educ. 2017;6:1916–7822. doi: 10.21083/ajote.v6i1.3970

[33] Labster Molecular Cloning Virtual Lab. https://www.labster.com/simulations/molecular-cloning/. Accessed January 13, 2021.

[34] Simmons A, Larios-Sanz M, Amin S, Rosell R. Using mini-reports to teach scientific writing to biology students. Am Biol Teach. 2014;76:551–555. doi: 10.1525/abt.2014.76.8.9

[35] Morris SB. Estimating effect sizes from pretest-posttest-control group designs. Organ Res Methods. 2008;11:364–386.

[36] Heckman JJ, Stixrud J, Urzua S. The effects of cognitive and noncognitive abilities on labor market outcomes and social behavior. J Labor Econ. 2006;24:411–482. doi: 10.1086/504455

[37] Walker JP, Sampson V, Grooms J, Zimmerman C. A performance-based assessment for limiting reactants. J Chem Educ. 2011;88:1243.

