# Supplementary material for: The effectiveness of scenario-based virtual laboratory simulations to improve learning outcomes and scientific report writing skills
Source: PLoS One. 2022 Nov 11;17(11):e0277359. doi: 10.1371/journal.pone.0277359 (PMC9651557; doi:10.1371/journal.pone.0277359)
Supplement: S1 Text — (DOCX) [file pone.0277359.s001.docx]

**S1 text.  The questionnaire used in the study for intrinsic motivation, self-efficacy, and knowledge**

**Intrinsic motivation (3 items)**

*Response options: Completely disagree, Disagree, Neutral, Agree, Completely Agree*

**Q1.** I enjoy working with the molecular biology course

**Q2.** Molecular biology activities are fun to perform

**Q3**. I would describe Molecular biology as very interesting

**Self-efficacy (8 items)**

*Response options: Completely disagree, Disagree, Neutral, Agree, Completely Agree*

**Q1.**I am confident and can understand the basic concepts of the molecular biology course

**Q2.**I am confident that I understand the most complex concepts related to molecular biology course

**Q3.**I am confident that I can do an excellent job on the assignments and tests in the molecular biology exercises

**Q4.**I expect to do well in the molecular biology course

**Q5.**I am confident that I can master the skills being taught in the molecular biology course

**Q6.**I believe I will receive an excellent grade in the molecular biology course

**Q7.**I’m optimistic I can understand the most challenging material presented in this course

**Q8.**Considering the difficulty of this course, the lecturer, and my skills, I think I will do well in the class

**Knowledge scale (molecular cloning) (6 items)**

*The cursive response in italic bold is the correct answer.*

***Q1.Which of the following enzyme is required for the end-to-end joining of DNA?***

- ***DNA ligase***
- Restriction endonuclease
- RNA polymerase
- DNA polymerase

***Q2. Which of the following techniques will we use to insert exogenous DNA into the E.coli?***

- ***Transformation***
- Transduction
- Conjugation
- All of the options

***Q3.Type II restriction endonucleases are useful in recombinant DNA research primarily because***

- ***They cut both strands of DNA at a specific recognition site.***
- They can distinguish between genomic and cDNA
- They can join two pieces of DNA by forming phosphodiester bonds.
- They cleave DNA at a distance from the recognition site.

***Q4. In recombinant DNA technology, the term competency refers to***

- The ability of DNA ends to be ligated
- The ability of an enzyme to be activated by ATP
- ***The ability of cells to take up foreign DNA***
- The ability of a medium to select for transformed cells

***Q5. Which of the following would NOT be a useful selectable marker?***

- ***A gene encoding a protein that degrades the antibiotic ampicillin.***
- A gene encoding a protein that allows the cell to synthesize histidine.
- A gene encoding a protein that is an essential structural component of the cell.
- All of these are useful selectable markers.

***Q6. Formation of what type of chemical bond is catalyzed by DNA ligase?***

- Glycosidic.
- Hydrogen.
- ***Phosphodiester bond***
- Ester.
