# Supplementary material for: The effectiveness of scenario-based virtual laboratory simulations to improve learning outcomes and scientific report writing skills
Source: PLoS One. 2022 Nov 11;17(11):e0277359. doi: 10.1371/journal.pone.0277359 (PMC9651557; doi:10.1371/journal.pone.0277359)
Supplement: S2 Text — (DOCX) [file pone.0277359.s002.docx]

**S10 text. Guidance for writing lab reports**

**Writing lab reports**

To write a successful scientific report you need to be clear about what you are trying to achieve. The main purpose of a scientific report is to communicate the finding from the work and to help the reader to understand them. The report should include a record of the process used to establish the findings, so they can be reproduced at a later stage for validation. It should be written as an independent record that can be read without further input from the author.

A typical scientific report should document what has been done, how it was done, what the findings were, and the author’s interpretation of those findings. A story should be told through a logical delivery of information. A molecular biology lab report should be presented in logical sections. The structure of these sections and style of presentation has evolved to convey essential information as concisely and effectively as possible.

**I. Title**

A molecular biology lab report may include a title page, contents page (with page numbers), list of tables, and list of figures.

**II. Introduction**

The introduction provides the reader with the background to the work documented in the report. This section should set the scene for what is to follow. It should contain the aims or objectives of the proposed work. If an aim of the experiment is to investigate a hypothesis, then this should be stated in the introduction. The aims, objectives and/or hypothesis should be given in the context of the real world application outside the experiment.

Begin with an opening paragraph stating what you are doing and what is the objective of these experiments or virtual lab simulations. Conclude the introduction with specific predictions of your expected results and how these results will answer your questions or support/refute your hypotheses.

In summary, the introduction should include:

- a background to the subject
- previously conduced work in the same subject
- aims and objectives for the work that will be presented in the lab report
- reasons why the work is being conducted

**III. Methods**

The procedure section is a record of what was done, a chronological description of the steps followed and the equipment used. It should not be a list of instructions but should be written as prose, in the third person and past tense (as should the rest of the report). Details of what variables were recorded, what observations were made, and what types of instrumentation were used should be included.

A well-written procedure should include not only a description of what was performed, but also the reasoning behind the experimental design. Why was the experiment set up in the way it was and how does it conform to the scientific method? What special measures have been put in place to ensure accuracy and repeatability of the results?

Additionally, draw a flow diagram shows all the virtual lab parts and how they connect with each other (e.g., the DNA goes from the restriction digest to the ligation to the gel). You do not need to show the amounts; just show what goes into each tube. In a flow diagram, all the components must connect with each other by a sequence of arrows.

**IV. Results**

The results section of a lab report contains an impartial description of the results obtained from the experiment, typically presented as tables or graphs, and observations that were made. At this point in the report, interpretation of the results should not be performed. To convey the main findings of the experiment, processed, rather than raw data, should be shown. The data presented in the results section should demonstrate how the experiment’s objectives have been met.

**V. Discussion**

This section is a discussion of what your results mean. The discussion format is typically the reverse of the introduction format: begin with specific details about your results and end with a general concluding statement. Finish the discussion with an overall summary.

**VI**. **References**

Include at least two references relevant to your findings
