# Supplementary material for: The effectiveness of scenario-based virtual laboratory simulations to improve learning outcomes and scientific report writing skills
Source: PLoS One. 2022 Nov 11;17(11):e0277359. doi: 10.1371/journal.pone.0277359 (PMC9651557; doi:10.1371/journal.pone.0277359)
Supplement: S1 Table — A-D. Percentage of student responses and Cronbach’s alpha calculation of student responses on the questionnaire of the self-efficacy (pre and post-test /control group). (DOCX) [file pone.0277359.s003.docx]

| **S2ATable.Cronbach's alpha calculation of student responses on the questionnaire of the self-efficacy (pre-test/control group, n=17)** | | | | | | | | | | |
| --- | --- | --- | --- | --- | --- | --- | --- | --- | --- | --- |
|  |  |  |  |  |  |  |  |  |  |  |
|  | | | | | | | | | | |
| **Students No** | **Q1** | **Q2** | **Q3** | **Q4** | **Q5** | **Q6** | **Q7** | **Q8** |  | **Overall** |
| **1** | 4 | 3 | 3 | 2 | 3 | 1 | 3 | 2 |  | 21 |
| **2** | 3 | 2 | 3 | 3 | 4 | 3 | 2 | 1 |  | 21 |
| **3** | 5 | 4 | 2 | 2 | 3 | 3 | 2 | 2 |  | 23 |
| **4** | 5 | 3 | 3 | 2 | 3 | 2 | 3 | 3 |  | 24 |
| **5** | 5 | 2 | 2 | 3 | 3 | 3 | 3 | 1 |  | 22 |
| **6** | 4 | 2 | 2 | 2 | 3 | 2 | 3 | 3 |  | 21 |
| **7** | 3 | 3 | 3 | 4 | 3 | 1 | 2 | 2 |  | 21 |
| **8** | 4 | 3 | 3 | 4 | 4 | 3 | 2 | 3 |  | 26 |
| **9** | 5 | 4 | 4 | 4 | 5 | 4 | 5 | 4 |  | 35 |
| **10** | 3 | 3 | 4 | 3 | 3 | 1 | 3 | 3 |  | 23 |
| **11** | 5 | 2 | 5 | 4 | 5 | 3 | 3 | 3 |  | 30 |
| **12** | 4 | 4 | 4 | 3 | 4 | 3 | 2 | 3 |  | 27 |
| **13** | 3 | 3 | 4 | 4 | 3 | 3 | 1 | 2 |  | 23 |
| **14** | 3 | 4 | 5 | 3 | 2 | 3 | 2 | 3 |  | 25 |
| **15** | 4 | 4 | 5 | 4 | 3 | 2 | 3 | 4 |  | 29 |
| **16** | 5 | 3 | 5 | 4 | 3 | 2 | 3 | 4 |  | 29 |
| **17** | 5 | 5 | 4 | 4 | 3 | 3 | 3 | 3 |  | 30 |
|  | 0.6920 | 0.7335 | 1.0657 | 0.6505 | 0.5813 | 0.7197 | 0.6989 | 0.7958 | 5.9377 | 15.9723 |
|  |  |  |  |  |  |  |  |  | **Cronbach's alpha** | **0.7179** |

**S2B Table. Percentage of student responses on the questionnaire of the self-efficacy recording student perceptions (pre-test/control group, n = 17)**

|  |  | **Likert Scale** | | | | | | | | | | | | |
| --- | --- | --- | --- | --- | --- | --- | --- | --- | --- | --- | --- | --- | --- | --- |
|  | **Completely Disagree** | | **Disagree** | | **Neutral** | | | **Agree** | | **Completely Agree** | |  |  |  |
| **Pre-test** | **1** | **%** | **2** | **%** | | **3** | **%** | **4** | **%** | **5** | **%** | **total** | **Weighted average** | |
| **Q1** | 0 | 0 | 0 | 0 | | 5 | 29.4117 | 5 | 29.4117 | 7 | 41.1764 | 70 | 4.1176 | |
| **Q2** | 0 | 0 | 4 | 23.5294 | | 7 | 41.1764 | 5 | 29.4117 | 1 | 5.8823 | 54 | 3.1764 | |
| **Q3** | 0 | 0 | 3 | 17.6470 | | 5 | 29.4117 | 5 | 29.4117 | 4 | 23.5294 | 61 | 3.5882 | |
| **Q4** | 0 | 0 | 4 | 23.5294 | | 5 | 29.4117 | 8 | 47.0588 | 0 | 0 | 55 | 3.2352 | |
| **Q5** | 0 | 0 | 1 | 5.8823 | | 11 | 64.7058 | 3 | 17.6470 | 2 | 11.7647 | 57 | 3.3529 | |
| **Q6** | 3 | 17.6470 | 4 | 23.52941 | | 9 | 52.9411 | 1 | 5.8823 | 0 | 0 | 42 | 2.4705 | |
| **Q7** | 1 | 5.8823 | 6 | 35.2941 | | 9 | 52.9411 | 0 | 0 | 1 | 5.8823 | 45 | 2.6470 | |
| **Q8** | 2 | 11.7647 | 4 | 23.5294 | | 8 | 47.0588 | 3 | 17.6470 | 0 | 0 | 46 | 2.7058 | |
|  |  |  |  |  | |  |  |  |  |  |  |  | **3.1617** | |

| **S2CTable. Cronbach's alpha calculation of student responses on the questionnaire of the self-efficacy (post-test/control group, n=17)** | | | | | | | | | | |
| --- | --- | --- | --- | --- | --- | --- | --- | --- | --- | --- |
|  |  |  |  |  |  |  |  |  |  |  |
|  | | | | | | | | | | |
| **Students No** | **Q1** | **Q2** | **Q3** | **Q4** | **Q5** | **Q6** | **Q7** | **Q8** |  | **Overall** |
| **1** | 4 | 2 | 3 | 2 | 3 | 2 | 3 | 2 |  | 21 |
| **2** | 3 | 2 | 3 | 3 | 4 | 3 | 2 | 4 |  | 24 |
| **3** | 4 | 3 | 2 | 2 | 4 | 3 | 3 | 2 |  | 23 |
| **4** | 5 | 3 | 3 | 2 | 3 | 2 | 3 | 3 |  | 24 |
| **5** | 5 | 2 | 2 | 3 | 3 | 3 | 3 | 4 |  | 25 |
| **6** | 3 | 2 | 2 | 3 | 3 | 2 | 3 | 3 |  | 21 |
| **7** | 3 | 3 | 3 | 4 | 3 | 3 | 2 | 2 |  | 23 |
| **8** | 4 | 3 | 3 | 4 | 4 | 3 | 2 | 3 |  | 26 |
| **9** | 5 | 4 | 4 | 4 | 5 | 4 | 5 | 5 |  | 36 |
| **10** | 3 | 4 | 3 | 3 | 3 | 2 | 2 | 3 |  | 23 |
| **11** | 5 | 2 | 5 | 4 | 5 | 3 | 3 | 3 |  | 30 |
| **12** | 4 | 4 | 4 | 3 | 2 | 4 | 3 | 3 |  | 27 |
| **13** | 3 | 3 | 4 | 4 | 3 | 3 | 1 | 2 |  | 23 |
| **14** | 3 | 4 | 5 | 3 | 3 | 3 | 2 | 3 |  | 26 |
| **15** | 4 | 4 | 5 | 4 | 3 | 2 | 3 | 4 |  | 29 |
| **16** | 5 | 3 | 5 | 4 | 3 | 2 | 3 | 4 |  | 29 |
| **17** | 5 | 5 | 4 | 4 | 4 | 3 | 3 | 3 |  | 31 |
|  | 0.7058 | 0.8096 | 1.0726 | 0.5605 | 0.5951 | 0.4152 | 0.6782 | 0.6920 | 5.5294 | 14.9965 |
|  |  |  |  |  |  |  |  |  | **Cronbach's alpha** | **0.7214** |

**S2D Table. Percentage of student responses on the questionnaire of the self-efficacy recording student perceptions (post-test/control group, n = 17)**

|  |  | **Likert Scale** | | | | | | | | | | | | |
| --- | --- | --- | --- | --- | --- | --- | --- | --- | --- | --- | --- | --- | --- | --- |
|  | **Completely Disagree** | | **Disagree** | | **Neutral** | | | **Agree** | | **Completely Agree** | |  |  |  |
| **Post-test** | **1** | **%** | **2** | **%** | | **3** | **%** | **4** | **%** | **5** | **%** | **total** | **Weighted average** | |
| **Q1** | 0 | 0 | 0 | 0 | | 6 | 35.2941 | 5 | 29.41176 | 6 | 35.2941 | 68 | 4 | |
| **Q2** | 0 | 0 | 5 | 29.4117 | | 6 | 35.2941 | 5 | 29.41176 | 1 | 5.8823 | 53 | 3.1176 | |
| **Q3** | 0 | 0 | 3 | 17.6470 | | 6 | 35.2941 | 4 | 23.5294 | 4 | 23.5294 | 60 | 3.5294 | |
| **Q4** | 0 | 0 | 3 | 17.6470 | | 6 | 35.29412 | 8 | 47.0588 | 0 | 0 | 56 | 3.2941 | |
| **Q5** | 0 | 0 | 1 | 5.8823 | | 10 | 58.82353 | 4 | 23.5294 | 2 | 11.7647 | 58 | 3.4117 | |
| **Q6** | 0 | 0 | 6 | 35.2941 | | 9 | 52.94118 | 2 | 11.7647 | 0 | 0 | 47 | 2.7647 | |
| **Q7** | 1 | 5.8823 | 5 | 29.4117 | | 10 | 58.82353 | 0 | 0 | 1 | 5.8823 | 46 | 2.7058 | |
| **Q8** | 0 | 0 | 4 | 23.5294 | | 8 | 47.05882 | 4 | 23.5294 | 1 | 5.8823 | 53 | 3.1176 | |
|  |  |  |  |  | |  |  |  |  |  |  |  | **3.2426** | |
