# Supplementary material for: The effectiveness of scenario-based virtual laboratory simulations to improve learning outcomes and scientific report writing skills
Source: PLoS One. 2022 Nov 11;17(11):e0277359. doi: 10.1371/journal.pone.0277359 (PMC9651557; doi:10.1371/journal.pone.0277359)
Supplement: S2 Table — A-D. Percentage of student responses and Cronbach’s alpha calculation of student responses on the questionnaire of the academic intrinsic motivation (pre and post-test /control group). (DOCX) [file pone.0277359.s004.docx]

**S3A Table. Cronbach's alpha calculation of student responses on the questionnaire of the academic intrinsic motivation (pre-test/control group, n=17)**

|  |  |  |  |  |  |
| --- | --- | --- | --- | --- | --- |
| **Students No** | **Q1** | **Q2** | **Q3** |  | **Overall** |
| 1 | 2 | 3 | 2 |  | 7 |
| 2 | 2 | 3 | 3 |  | 8 |
| 3 | 3 | 4 | 4 |  | 11 |
| 4 | 1 | 2 | 2 |  | 5 |
| 5 | 1 | 3 | 4 |  | 8 |
| 6 | 3 | 3 | 3 |  | 9 |
| 7 | 2 | 3 | 2 |  | 7 |
| 8 | 3 | 2 | 2 |  | 7 |
| 9 | 2 | 2 | 3 |  | 7 |
| 10 | 1 | 1 | 3 |  | 5 |
| 11 | 3 | 2 | 3 |  | 8 |
| 12 | 3 | 3 | 4 |  | 10 |
| 13 | 3 | 2 | 3 |  | 8 |
| 14 | 4 | 5 | 4 |  | 13 |
| 15 | 5 | 4 | 5 |  | 14 |
| 16 | 4 | 4 | 4 |  | 12 |
| 17 | 2 | 1 | 3 |  | 6 |
|  | 1.1834 | 1.12114 | 0.7336 | 3.0381 | 6.6021 |
|  |  |  |  | **Cronbach's alpha** | **0.8097** |

**S3B Table. Percentage of student responses on the questionnaire of the academic intrinsic motivation recording student perceptions (pre-test/control group, n = 17)**

|  |  | **Likert Scale** | | | | | | | | | | | | |
| --- | --- | --- | --- | --- | --- | --- | --- | --- | --- | --- | --- | --- | --- | --- |
|  | **Completely Disagree** | | **Disagree** | | **Neutral** | | | **Agree** | | **Completely Agree** | |  |  |  |
| **Pre-test** | **1** | **%** | **2** | **%** | | **3** | **%** | **4** | **%** | **5** | **%** | **total** | **Weighted average** | |
| **Q1** | 3 | 17.6471 | 5 | 29.4117 | | 6 | 35.2941 | 1 | 5.8823 | 1 | 5.8823 | 40 | 2.3529 | |
| **Q2** | 2 | 11.7647 | 6 | 35.2941 | | 6 | 35.2941 | 3 | 17.6471 | 1 | 5.8823 | 49 | 2.8823 | |
| **Q3** | 0 | 0 | 4 | 23.52941 | | 7 | 41.1765 | 5 | 29.4117 | 1 | 5.8823 | 54 | 3.1765 | |
|  |  |  |  |  | |  |  |  |  |  |  |  | **2.8039** | |

**S3C Table. Cronbach's alpha calculation of student responses on the questionnaire of the academic intrinsic motivation (post-test/control group, n=17)**

|  |  |  |  |  |  |
| --- | --- | --- | --- | --- | --- |
| **Students No** | **Q1** | **Q2** | **Q3** |  | **Overall** |
| **1** | 2 | 3 | 3 |  | 8 |
| **2** | 2 | 2 | 2 |  | 6 |
| **3** | 2 | 2 | 3 |  | 7 |
| **4** | 2 | 2 | 3 |  | 7 |
| **5** | 4 | 4 | 4 |  | 12 |
| **6** | 2 | 1 | 3 |  | 6 |
| **7** | 5 | 4 | 4 |  | 13 |
| **8** | 5 | 4 | 4 |  | 13 |
| **9** | 3 | 2 | 3 |  | 8 |
| **10** | 3 | 3 | 4 |  | 10 |
| **11** | 3 | 2 | 3 |  | 8 |
| **12** | 2 | 3 | 3 |  | 8 |
| **13** | 3 | 4 | 3 |  | 10 |
| **14** | 3 | 2 | 3 |  | 8 |
| **15** | 3 | 2 | 2 |  | 7 |
| **16** | 2 | 3 | 3 |  | 8 |
| **17** | 4 | 3 | 3 |  | 10 |
|  | 0.9965 | 0.7958 | 0.3391 | 2.1315 | 4.6505 |
|  |  |  |  | **Cronbach's alpha** | **0.8125** |

**S3D Table. Percentage of student responses on the questionnaire of the academic intrinsic motivation recording student perceptions (post-test/control group, n = 17)**

|  |  | **Likert Scale** | | | | | | | | | | | | |
| --- | --- | --- | --- | --- | --- | --- | --- | --- | --- | --- | --- | --- | --- | --- |
|  | **Completely Disagree** | | **Disagree** | | **Neutral** | | | **Agree** | | **Completely Agree** | |  |  |  |
| **Post-test** | **1** | **%** | **2** | **%** | | **3** | **%** | **4** | **%** | **5** | **%** | **total** | **Weighted average** | |
| **Q1** | 0 | 0 | 7 | 41.1765 | | 6 | 35.2941 | 2 | 11.7647 | 5 | 29.41176 | 65 | 3.8235 | |
| **Q2** | 1 | 5.8823 | 7 | 41.1765 | | 5 | 29.4118 | 4 | 23.5294 | 0 | 0 | 46 | 2.7058 | |
| **Q3** | 0 | 0 | 2 | 11.7647 | | 11 | 64.7059 | 4 | 23.5294 | 0 | 0 | 53 | 3.1176 | |
|  |  |  |  |  | |  |  |  |  |  |  |  | **3.2157** | |
