# Supplementary material for: The effectiveness of scenario-based virtual laboratory simulations to improve learning outcomes and scientific report writing skills
Source: PLoS One. 2022 Nov 11;17(11):e0277359. doi: 10.1371/journal.pone.0277359 (PMC9651557; doi:10.1371/journal.pone.0277359)
Supplement: S3 Table — A-D. Percentage of student responses and Cronbach’s alpha calculation of student responses on the questionnaire of the self-efficacy (pre and post-test / experimental group). (DOCX) [file pone.0277359.s005.docx]

| **S4ATable.Cronbach's alpha calculation of student responses on the questionnaire of the self-efficacy (pre-test/** **experimental group,n=18)** | | | | | | | | | | |
| --- | --- | --- | --- | --- | --- | --- | --- | --- | --- | --- |
|  |  |  |  |  |  |  |  |  |  |  |
|  | | | | | | | | | | |
| **Students No** | **Q1** | **Q2** | **Q3** | **Q4** | **Q5** | **Q6** | **Q7** | **Q8** |  | **Overall** |
| **1** | 3 | 3 | 3 | 4 | 3 | 1 | 2 | 2 |  | 21 |
| **2** | 3 | 3 | 3 | 4 | 4 | 3 | 2 | 3 |  | 21 |
| **3** | 3 | 4 | 5 | 3 | 2 | 3 | 2 | 3 |  | 23 |
| **4** | 4 | 4 | 5 | 4 | 3 | 2 | 3 | 4 |  | 23 |
| **5** | 4 | 2 | 2 | 3 | 3 | 3 | 3 | 1 |  | 21 |
| **6** | 4 | 3 | 3 | 2 | 3 | 2 | 3 | 3 |  | 23 |
| **7** | 3 | 3 | 3 | 4 | 3 | 1 | 2 | 2 |  | 21 |
| **8** | 4 | 3 | 3 | 4 | 4 | 3 | 2 | 3 |  | 26 |
| **9** | 3 | 3 | 4 | 3 | 3 | 1 | 3 | 3 |  | 35 |
| **10** | 5 | 2 | 5 | 4 | 5 | 3 | 3 | 3 |  | 23 |
| **11** | 4 | 4 | 4 | 3 | 4 | 3 | 2 | 3 |  | 30 |
| **12** | 3 | 3 | 4 | 4 | 3 | 3 | 1 | 2 |  | 27 |
| **13** | 5 | 4 | 4 | 4 | 5 | 4 | 5 | 4 |  | 23 |
| **14** | 4 | 3 | 3 | 2 | 3 | 1 | 3 | 2 |  | 25 |
| **15** | 3 | 2 | 3 | 3 | 4 | 3 | 2 | 1 |  | 29 |
| **16** | 5 | 3 | 5 | 4 | 3 | 2 | 3 | 4 |  | 29 |
| **17** | 5 | 5 | 4 | 4 | 3 | 3 | 3 | 3 |  | 30 |
| **18** | 5 | 4 | 2 | 2 | 3 | 3 | 2 | 2 |  | 23 |
|  | 0.6543 | 0.6172 | 0.9043 | 0.5709 | 0.5709 | 0.8024 | 0.6913 | 0.7777 | 4.8117 | 15.25 |
|  |  |  |  |  |  |  |  |  | **Cronbach's alpha** | **0.7822** |

**S4B Table. Percentage of student responses on the questionnaire of the self-efficacy recording student perceptions (pre-test/ experimental group, n = 18)**

|  |  | **Likert Scale** | | | | | | | | | | | | |
| --- | --- | --- | --- | --- | --- | --- | --- | --- | --- | --- | --- | --- | --- | --- |
|  | **Completely Disagree** | | **Disagree** | | **Neutral** | | | **Agree** | | **Completely Agree** | |  |  |  |
|  | **1** | **%** | **2** | **%** | | **3** | **%** | **4** | **%** | **5** | **%** | **total** | **Weighted average** | |
| **Q1** | 0 | 0 | 0 | 0 | | 7 | 38.88889 | 6 | 33.3333 | 5 | 27.7778 | 70 | 4.1176 | |
| **Q2** | 0 | 0 | 3 | 16.6666 | | 9 | 50 | 5 | 27.7778 | 1 | 5.5556 | 58 | 3.4117 | |
| **Q3** | 0 | 0 | 2 | 11.1111 | | 7 | 38.8889 | 5 | 27.7778 | 4 | 22.2222 | 65 | 3.8235 | |
| **Q4** | 0 | 0 | 3 | 16.6666 | | 5 | 27.7778 | 10 | 55.5556 | 0 | 0 | 61 | 3.5882 | |
| **Q5** | 0 | 0 | 1 | 5.5555 | | 11 | 61.1111 | 4 | 22.2222 | 2 | 11.1111 | 61 | 3.5882 | |
| **Q6** | 4 | 22.2222 | 3 | 16.6666 | | 10 | 55.5555 | 1 | 5.5556 | 0 | 0 | 44 | 2.5882 | |
| **Q7** | 1 | 5.55555 | 8 | 44.4444 | | 8 | 44.4444 | 0 | 0 | 1 | 5.5556 | 46 | 2.7058 | |
| **Q8** | 2 | 11.1111 | 5 | 27.7777 | | 8 | 44.4444 | 3 | 16.6667 | 0 | 0 | 48 | 2.8235 | |
|  |  |  |  |  | |  |  |  |  |  |  |  | **3.33088** | |

| **S4C Table. Cronbach's alpha calculation of student responses on the questionnaire of the self-efficacy (post-test/ experimental group, n=18)** | | | | | | | | | | |
| --- | --- | --- | --- | --- | --- | --- | --- | --- | --- | --- |
|  |  |  |  |  |  |  |  |  |  |  |
|  | | | | | | | | | | |
| **Students No** | **Q1** | **Q2** | **Q3** | **Q4** | **Q5** | **Q6** | **Q7** | **Q8** |  | **Overall** |
| **1** | 4 | 4 | 5 | 4 | 4 | 3 | 4 | 4 |  | 32 |
| **2** | 5 | 3 | 5 | 4 | 4 | 4 | 4 | 4 |  | 33 |
| **3** | 5 | 4 | 3 | 3 | 3 | 4 | 3 | 3 |  | 28 |
| **4** | 5 | 4 | 3 | 3 | 3 | 3 | 3 | 4 |  | 28 |
| **5** | 4 | 3 | 3 | 4 | 4 | 3 | 4 | 3 |  | 28 |
| **6** | 5 | 4 | 4 | 5 | 4 | 4 | 5 | 5 |  | 36 |
| **7** | 3 | 3 | 3 | 3 | 4 | 3 | 2 | 1 |  | 22 |
| **8** | 4 | 3 | 3 | 4 | 2 | 3 | 3 | 2 |  | 24 |
| **9** | 4 | 4 | 4 | 5 | 5 | 4 | 3 | 3 |  | 32 |
| **10** | 4 | 5 | 4 | 4 | 4 | 3 | 2 | 2 |  | 28 |
| **11** | 4 | 2 | 3 | 3 | 3 | 4 | 4 | 3 |  | 26 |
| **12** | 3 | 3 | 4 | 4 | 4 | 4 | 2 | 2 |  | 26 |
| **13** | 3 | 4 | 3 | 3 | 4 | 4 | 3 | 3 |  | 27 |
| **14** | 5 | 3 | 5 | 4 | 5 | 3 | 3 | 3 |  | 31 |
| **15** | 5 | 5 | 4 | 4 | 3 | 4 | 3 | 3 |  | 31 |
| **16** | 4 | 4 | 5 | 5 | 4 | 3 | 4 | 3 |  | 32 |
| **17** | 5 | 5 | 4 | 4 | 4 | 3 | 4 | 3 |  | 32 |
| **18** | 4 | 3 | 3 | 3 | 3 | 4 | 4 | 3 |  | 27 |
|  | 0.5061 | 0.6667 | 0.6172 | 0.4722 | 0.5339 | 0.25 | 0.6667 | 0.7778 | 4.4907 | 11.8302 |
|  |  |  |  |  |  |  |  |  | **Cronbach's alpha** | **0.7090** |

**S4D Table. Percentage of student responses on the questionnaire of the self-efficacy recording student perceptions (post-test/ experimental group, n = 18)**

|  |  | **Likert Scale** | | | | | | | | | | | | |
| --- | --- | --- | --- | --- | --- | --- | --- | --- | --- | --- | --- | --- | --- | --- |
|  | **Completely Disagree** | | **Disagree** | | **Neutral** | | | **Agree** | | **Completely Agree** | |  |  |  |
|  | **1** | **%** | **2** | **%** | | **3** | **%** | **4** | **%** | **5** | **%** | **total** | **Weighted average** | |
| **Q1** | 0 | 0 | 0 | 0 | | 3 | 16.6667 | 8 | 44.4444 | 7 | 38.8889 | 76 | 4.4706 | |
| **Q2** | 0 | 0 | 1 | 5.5556 | | 7 | 38.8889 | 7 | 38.8889 | 3 | 16.6667 | 66 | 3.8823 | |
| **Q3** | 0 | 0 | 0 | 0 | | 8 | 44.4444 | 6 | 33.3333 | 4 | 22.2222 | 68 | 4 | |
| **Q4** | 0 | 0 | 0 | 0 | | 6 | 33.3333 | 9 | 50 | 3 | 16.6667 | 69 | 4.0588 | |
| **Q5** | 0 | 0 | 1 | 5.5556 | | 5 | 27.7778 | 10 | 55.5556 | 2 | 11.1111 | 67 | 3.9412 | |
| **Q6** | 0 | 0 | 0 | 0 | | 9 | 50 | 9 | 50 | 0 | 0 | 63 | 3.7059 | |
| **Q7** | 0 | 0 | 3 | 16.6667 | | 7 | 38.8889 | 7 | 38.8889 | 1 | 5.5556 | 60 | 3.5294 | |
| **Q8** | 1 | 5.5556 | 3 | 16.6667 | | 10 | 55.55556 | 3 | 16.6667 | 1 | 5.5556 | 54 | 3.1764 | |
|  |  |  |  |  | |  |  |  |  |  |  |  | **3.8456** | |
