# Supplementary material for: The effectiveness of scenario-based virtual laboratory simulations to improve learning outcomes and scientific report writing skills
Source: PLoS One. 2022 Nov 11;17(11):e0277359. doi: 10.1371/journal.pone.0277359 (PMC9651557; doi:10.1371/journal.pone.0277359)
Supplement: S4 Table — A-D. Percentage of student responses and Cronbach’s alpha calculation of student responses on the questionnaire of the academic intrinsic motivation (pre and post-test / experimental group). (DOCX) [file pone.0277359.s006.docx]

**S5A Table. Cronbach's alpha calculation of student responses on the questionnaire of the academic intrinsic motivation (pre-test/ experimental group, n=18)**

|  |  |  |  |  |  |
| --- | --- | --- | --- | --- | --- |
| **Students No** | **Q1** | **Q2** | **Q3** |  | **Overall** |
| 1 | 2 | 2 | 2 |  | 6 |
| 2 | 3 | 4 | 5 |  | 12 |
| 3 | 1 | 1 | 3 |  | 5 |
| 4 | 2 | 3 | 3 |  | 8 |
| 5 | 2 | 2 | 2 |  | 6 |
| 6 | 2 | 2 | 3 |  | 7 |
| 7 | 1 | 2 | 2 |  | 5 |
| 8 | 3 | 3 | 3 |  | 9 |
| 9 | 2 | 2 | 3 |  | 7 |
| 10 | 2 | 3 | 3 |  | 8 |
| 11 | 2 | 3 | 3 |  | 8 |
| 12 | 1 | 3 | 2 |  | 6 |
| 13 | 3 | 2 | 3 |  | 8 |
| 14 | 3 | 3 | 4 |  | 10 |
| 15 | 4 | 5 | 4 |  | 13 |
| 16 | 3 | 2 | 3 |  | 8 |
| 17 | 4 | 4 | 3 |  | 11 |
| 18 | 4 | 3 | 4 |  | 11 |
|  | 0.9136 | 0.8673 | 0.6080 | 2.3889 | 5.28395 |
|  |  |  |  | **Cronbach's alpha** | **0.8218** |

**S5B Table. Percentage of student responses on the questionnaire of the academic intrinsic motivation recording student perceptions (pre-test/ experimental group, n = 18)**

|  |  | **Likert Scale** | | | | | | | | | | | | |
| --- | --- | --- | --- | --- | --- | --- | --- | --- | --- | --- | --- | --- | --- | --- |
|  | **Completely Disagree** | | **Disagree** | | **Neutral** | | | **Agree** | | **Completely Agree** | |  |  |  |
| **Pre-test** | **1** | **%** | **2** | **%** | | **3** | **%** | **4** | **%** | **5** | **%** | **total** | **Weighted average** | |
| **Q1** | 3 | 16.6667 | 7 | 38.8889 | | 5 | 27.7778 | 3 | 16.6667 | 0 | 0 | 44 | 2.5882 | |
| **Q2** | 1 | 5.5556 | 7 | 38.8889 | | 7 | 38.8889 | 2 | 11.1111 | 1 | 5.5556 | 49 | 2.8823 | |
| **Q3** | 0 | 0 | 4 | 22.2222 | | 10 | 55.5556 | 3 | 16.6667 | 1 | 5.5556 | 55 | 3.2353 | |
|  |  |  |  |  | |  |  |  |  |  |  |  | **2.9019** | |

**S5C Table. Cronbach's alpha calculation of student responses on the questionnaire of the academic intrinsic motivation (post-test/** **experimental group, n=18)**

|  |  |  |  |  |  |
| --- | --- | --- | --- | --- | --- |
| **Students No** | **Q1** | **Q2** | **Q3** |  | **Overall** |
| **1** | 4 | 5 | 5 |  | 14 |
| **2** | 4 | 4 | 3 |  | 11 |
| **3** | 4 | 4 | 3 |  | 11 |
| **4** | 2 | 2 | 3 |  | 7 |
| **5** | 4 | 4 | 5 |  | 13 |
| **6** | 3 | 4 | 3 |  | 10 |
| **7** | 5 | 4 | 4 |  | 13 |
| **8** | 5 | 4 | 4 |  | 13 |
| **9** | 3 | 3 | 3 |  | 9 |
| **10** | 3 | 3 | 4 |  | 10 |
| **11** | 3 | 4 | 4 |  | 11 |
| **12** | 4 | 3 | 3 |  | 10 |
| **13** | 3 | 4 | 3 |  | 10 |
| **14** | 3 | 2 | 3 |  | 8 |
| **15** | 4 | 5 | 4 |  | 13 |
| **16** | 4 | 3 | 3 |  | 10 |
| **17** | 4 | 3 | 4 |  | 11 |
| **18** | 4 | 5 | 4 |  | 13 |
|  | 0.5556 | 0.7778 | 0.4598 | 1.7932 | 3.4969 |
|  |  |  |  | **Cronbach's alpha** | **0.7308** |

**S5D Table. Percentage of student responses on the questionnaire of the academic intrinsic motivation recording student perceptions (post-test/ experimental group, n=18)**

|  |  | **Likert Scale** | | | | | | | | | | | | |
| --- | --- | --- | --- | --- | --- | --- | --- | --- | --- | --- | --- | --- | --- | --- |
|  | **Completely Disagree** | | **Disagree** | | **Neutral** | | | **Agree** | | **Completely Agree** | |  |  |  |
| **Post-test** | **1** | **%** | **2** | **%** | | **3** | **%** | **4** | **%** | **5** | **%** | **total** | **Weighted average** | |
| **Q1** | 0 | 0 | 1 | 5.5556 | | 6 | 33.3333 | 9 | 50 | 2 | 11.1111 | 66 | 3.8823 | |
| **Q2** | 0 | 0 | 2 | 11.1111 | | 5 | 27.7778 | 8 | 44.44444 | 3 | 16.6667 | 66 | 3.8823 | |
| **Q3** | 0 | 0 | 0 | 0 | | 9 | 50 | 7 | 38.8889 | 2 | 11.1111 | 65 | 3.8235 | |
|  |  |  |  |  | |  |  |  |  |  |  |  | **3.8627** | |
