# Supplementary material for: The effectiveness of scenario-based virtual laboratory simulations to improve learning outcomes and scientific report writing skills
Source: PLoS One. 2022 Nov 11;17(11):e0277359. doi: 10.1371/journal.pone.0277359 (PMC9651557; doi:10.1371/journal.pone.0277359)
Supplement: S5 Table — (DOCX) [file pone.0277359.s007.docx]

| **S6Table.Comparisons in average % correct responses for knowledge scale (pre-posttest) for the SB-VLS and the VLD groups** | | | | |
| --- | --- | --- | --- | --- |
|  |  |  |  |  |
|  | **Experimental group (SB-VLS)** | | | |
| **n=18** |  | **Pre-test** |  | **Post-test** |
|  | **No of students with correct responses** | **% correct responses** | **No of students with correct responses** | **% correct responses** |
| **Q1** | **12** | 66.6667 | 17 | 94.4444 |
| **Q2** | **9** | 50 | 15 | 83.3333 |
| **Q3** | **7** | 38.8889 | 13 | 72.2222 |
| **Q4** | **8** | 44.4444 | 11 | 61.1111 |
| **Q5** | **5** | 27.7778 | 10 | 55.5556 |
| **Q6** | **14** | 77.7778 | 16 | 88.8889 |
| **Average % correct responses** |  | **50.9259** |  | **75.9259** |
|  |  |  |  |  |
|  | **Control group (VLD)** | | | |
| **n=17** |  | **Pre-test** |  | **Post-test** |
|  | **No of students with correct responses** | **% correct responses** | **No of students with correct responses** | **% correct responses** |
| **Q1** | **11** | 64.70588 | 12 | 70.5882 |
| **Q2** | **8** | 47.0588 | 11 | 64.7058 |
| **Q3** | **7** | 41.1765 | 9 | 52.9412 |
| **Q4** | **7** | 41.1765 | 8 | 47.0588 |
| **Q5** | **5** | 29.4117 | 5 | 29.4118 |
| **Q6** | **12** | 70.5882 | 12 | 70.5882 |
| **Average % correct responses** |  | **49.0196** |  | **55.8823** |

*SB-VLS, scenario-based virtual laboratory simulation; VLD, video lab demonstration*
