# Supplementary material for: The effectiveness of scenario-based virtual laboratory simulations to improve learning outcomes and scientific report writing skills
Source: PLoS One. 2022 Nov 11;17(11):e0277359. doi: 10.1371/journal.pone.0277359 (PMC9651557; doi:10.1371/journal.pone.0277359)
Supplement: S6 Table — (DOCX) [file pone.0277359.s008.docx]

| **S7Table. lab report scores results** | | |
| --- | --- | --- |
|  |  |  |
| **Students no** | **Control group**  **N=17** | **Experimental group**  **N=18** |
|  | **VDL** | **SB-VLS** |
| 1 | 4 | 4 |
| 2 | 4.5 | 4.5 |
| 3 | 3.75 | 4.5 |
| 4 | 3.5 | 5 |
| 5 | 4 | 4.75 |
| 6 | 3.6 | 5 |
| 7 | 4 | 5 |
| 8 | 4.75 | 5 |
| 9 | 3 | 4 |
| 10 | 4.75 | 4.75 |
| 11 | 4.5 | 5 |
| 12 | 4.5 | 5 |
| 13 | 4 | 4.5 |
| 14 | 3 | 4.5 |
| 15 | 3 | 5 |
| 16 | 3.75 | 4.5 |
| 17 | 4 | 5 |
| 18 | - | 5 |
| **Average lab report scores** | **3.918** | **4.722** |

*SB-VLS, scenario-based virtual laboratory simulation; VLD, video lab demonstration*
