# Supplementary material for: The effectiveness of scenario-based virtual laboratory simulations to improve learning outcomes and scientific report writing skills
Source: PLoS One. 2022 Nov 11;17(11):e0277359. doi: 10.1371/journal.pone.0277359 (PMC9651557; doi:10.1371/journal.pone.0277359)
Supplement: S7 Table — (DOCX) [file pone.0277359.s009.docx]

| **S8Table. Lab report grading rubric** |  |  |  |  |  |
| --- | --- | --- | --- | --- | --- |
|  |  |  |  |  |  |
| **Sections** | **Possible Points** | | | | |
|  | **A** | **B** | **C** | **D** | **F** |
| **1. Title (3 pts)** |  |  |  |  |  |
| Title is descriptive | 2 | 1.5 | 1 | 0.5 | 0 |
| Names of group members, professor, and section | 1 | 0.8 | 0.7 | 0.6 | 0 |
| **2. Introduction (10 pts)** |  |  |  |  |  |
| Background information (with relevant outside source) | 6 | 5 | 4 | 3 | 0 |
| Rationale/hypothesis | 4 | 3.5 | 2.5 | 2 | 0 |
| **3. Methods (8 pts)** |  |  |  |  |  |
| Experimental design (paragraph) | 5 | 4 | 3 | 2 | 0 |
| Correct format (paragraph, past tense, etc.) | 1 | 0.8 | 0.7 | 0.6 | 0 |
| Variables correctly identified, controls stated | 1 | 0.8 | 0.7 | 0.6 | 0 |
| Number of replicates stated | 1 | 0.8 | 0.7 | 0.6 | 0 |
| **4. Results (10 pts)** |  |  |  |  |  |
| Description of results | 5 | 4 | 3 | 2 | 0 |
| Figure pointed out in text | 1 | 0.8 | 0.7 | 0.6 | 0 |
| Correct type of figure | 2 | 1.5 | 1 | 0.5 | 0 |
| Correct labeling of figure/table (descriptive legend/title) | 2 | 1.5 | 1 | 0.5 | 0 |
| **5. Discussion and Conclusion (14 pts)** |  |  |  |  |  |
| Do the data support the hypothesis? | 3 | 2 | 1 .5 | 1 | 0 |
| Interpretation of results | 7 | 6 | 5 | 4 | 0 |
| Comparison of data with previously published information | 4 | 3.5 | 2.5 | 2 | 0 |
| **6. Literature Cited (5 pts)** |  |  |  |  |  |
| Literature is cited using the correct format (in text and in this section) | 2 | 1.5 | 1 | 0.5 | 0 |
| Good-quality, relevant citations | 2 | 1.5 | 1 | 0.5 | 0 |
| Good quantity of citations | 1 | 0.8 | 0.7 | 0.6 | 0 |
| **TOTAL (out of 50 pts)** |  |  |  |  |  |
|  |  |  |  |  |  |
| References: Simmons AD, Larios-Sanz M, Amin S, Rosell RC. Using mini-reports to teach scientific writing to biology students. Am Bio Teach. 2014;76: 551-555. doi: 10.1525/abt.2014.76.8.9. | | | | | |
