# Supplementary material for: The effectiveness of scenario-based virtual laboratory simulations to improve learning outcomes and scientific report writing skills
Source: PLoS One. 2022 Nov 11;17(11):e0277359. doi: 10.1371/journal.pone.0277359 (PMC9651557; doi:10.1371/journal.pone.0277359)
Supplement: S8 Table — (DOCX) [file pone.0277359.s010.docx]

**S9 Table. Reference to determine the Level of size Effect (²η) and (d)**

| **d** | **r^*^** | **η^2^** | **Interpretation sensu Cohen (1988)** | **Interpretation sensu Hattie (2009)** |
| --- | --- | --- | --- | --- |
| < 0 | < 0 | - | Adverse Effect | |
| 0 | 0 | 0 | No Effect | Developmental effects |
| 0.1 | 0.05 | 0.003 |  |  |
| 0.2 | 0.1 | 0.01 | Small Effect | Teacher effects |
| 0.3 | 0.15 | 0.022 |  |  |
| 0.4 | 0.2 | 0.039 |  | Zone of desired effects |
| 0.5 | 0.24 | 0.06 | Intermediate Effect |  |
| 0.6 | 0.29 | 0.083 |  |  |
| 0.7 | 0.33 | 0.11 |  |  |
| 0.8 | 0.37 | 0.14 | Large Effect |  |
| 0.9 | 0.41 | 0.168 |  |  |
| ≥ 1.0 | 0.45 | 0.2 |  |  |
|  |  |  |  |  |
| Reference:https://www.psychometrica.de/effect_size.html#transform | | | | |
