# Supplementary material for: The effectiveness of scenario-based virtual laboratory simulations to improve learning outcomes and scientific report writing skills
Source: PLoS One. 2022 Nov 11;17(11):e0277359. doi: 10.1371/journal.pone.0277359 (PMC9651557; doi:10.1371/journal.pone.0277359)
Supplement: S9 Table — (DOCX) [file pone.0277359.s011.docx]

**S11 Table. Power by effect size**


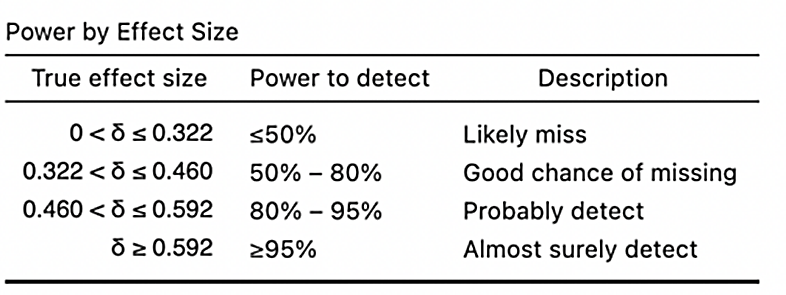


Reference: Bartlett J, Charles S. Power to the People: A Beginner’s Tutorial to Power Analysis using jamovi. 2021.
